# Continuous visualization and validation of pain in critically ill patients using artificial intelligence: a retrospective observational study

**DOI:** 10.1038/s41598-023-44970-2

**Published:** 2023-10-14

**Authors:** Naoya Kobayashi, Kazuki Watanabe, Hitoshi Murakami, Masanori Yamauchi

**Affiliations:** 1 Department of Anesthesiology and Perioperative Medicine, Tohoku University Graduate School of Medicine, Sendai, Miyagi Japan; 2 Hitachi Solutions East Japan, Ltd, Sendai, Miyagi Japan

**Keywords:** Medical research, Signs and symptoms

## Abstract

Machine learning tools have demonstrated viability in visualizing pain accurately using vital sign data; however, it remains uncertain whether incorporating individual patient baselines could enhance accuracy. This study aimed to investigate improving the accuracy by incorporating deviations from baseline patient vital signs and the concurrence of the predicted artificial intelligence values with the probability of critical care pain observation tool (CPOT) ≥ 3 after fentanyl administration. The study included adult patients in intensive care who underwent multiple pain-related assessments. We employed a random forest model, utilizing arterial pressure, heart rate, respiratory rate, gender, age, and Richmond Agitation–Sedation Scale score as explanatory variables. Pain was measured as the probability of CPOT scores of ≥ 3, and subsequently adjusted based on each patient's baseline. The study included 10,299 patients with 117,190 CPOT assessments. Of these, 3.3% had CPOT scores of ≥ 3. The random forest model demonstrated strong accuracy with an area under the receiver operating characteristic curve of 0.903. Patients treated with fentanyl were grouped based on CPOT score improvement. Those with ≥ 1-h of improvement after fentanyl administration had a significantly lower pain index (*P* = 0.020). Therefore, incorporating deviations from baseline patient vital signs improved the accuracy of pain visualization using machine learning techniques.

## Introduction

Many critically ill patients in the intensive care unit (ICU) experience intense pain regardless of whether they have undergone surgery^1–5^. Pain has a wide variety of adverse effects on patients, including psychological stress, sleep disturbances, decreased respiratory function, increased heart rate and blood pressure, arrhythmia, and poor nutritional status that can lead to prolonged hospital stays, increased treatment costs, and poor life outcomes^6–9^.

Although patient self-reporting is considered the most accurate and reliable way to assess pain, many critically ill patients are unable to self-evaluate due to intubation, tracheotomy, analgesic sedatives, or delirium; hence, alternative methods of assessing pain are necessary. Assessment scales, such as the Behavioral Pain Scale (BPS)^10^ and Critical Care Pain Observation Tool (CPOT, Supplementary Table S1)^11^, which are used^12–16^ as alternatives, are easy for observers to use and allow for some standardization of assessments; however, they are human evaluations, which may lead to inter-rater differences, albeit acceptable. Nevertheless, such differences may be eliminated if the evaluation is performed by a machine. Furthermore, since only intermittent assessments are possible, pain treatment may be delayed.

Machine learning analysis of vital sign data can overcome the shortcomings of conventional pain assessment methods and predict pain at a given time with extremely high accuracy^17^. The comprehensive analysis of time-series data conducted by Kobayashi et al.^17^ allowed three types of machine learning methods to predict pain with high accuracy, with the best random forest method achieving an area under the receiver operating characteristics curve (AUROC) of 0.85 for predicting whether the patient had a CPOT score of ≥ 3. However, individual differences in vital signs (e.g., variations in vascular, cardiac, and neurological functions) may affect the accuracy of pain prediction in conventional models, thereby reducing the prediction accuracy.

Therefore, the purpose of this study was to investigate (1) the possibility of improving the prediction accuracy by incorporating deviations from baseline patient vital signs into the prediction model and (2) the concurrence of the predicted artificial intelligence (AI) values with the scores obtained using the CPOT, a conventional objective pain assessment, during analgesic administration.

## Results

**Figure 1:**
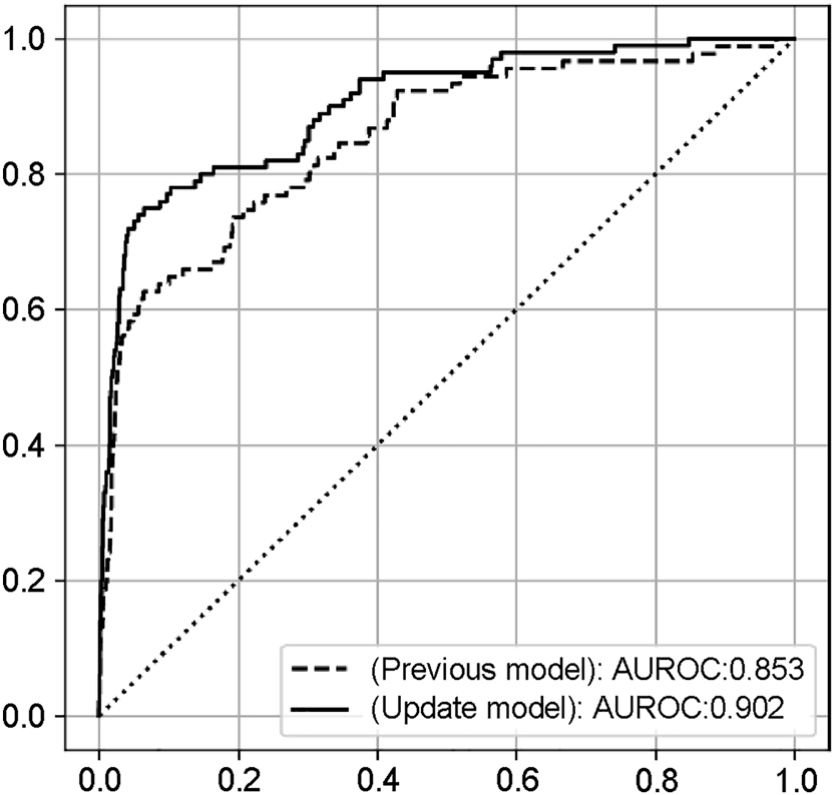
Accuracy of pain visualization. The “previous model” represents the previously reported model^17^, whereas the “updated model” represents the AUROC of the model proposed in this study. The x-axis and y-axis represent the negative sensitivity and specificity in the ROC curve, respectively. The accuracy of the test depends on the ability of the machine learning model to correctly determine whether the CPOT score is < 2 or > 3. The accuracy is expressed by AUROC, where a range of 1 indicates a perfect test and a range of 0.5 indicates an inconclusive test. In the previous model, the sensitivity was 64.8%, the specificity was 88.2%, the positive predictive value was 11.0%, and the negative predictive value was 99.1%. In the updated model, the sensitivity was 73.0%, the specificity was 94.5%, the positive predictive value was 28.0%, and the negative predictive value was 99.2%.

**Figure 2:**
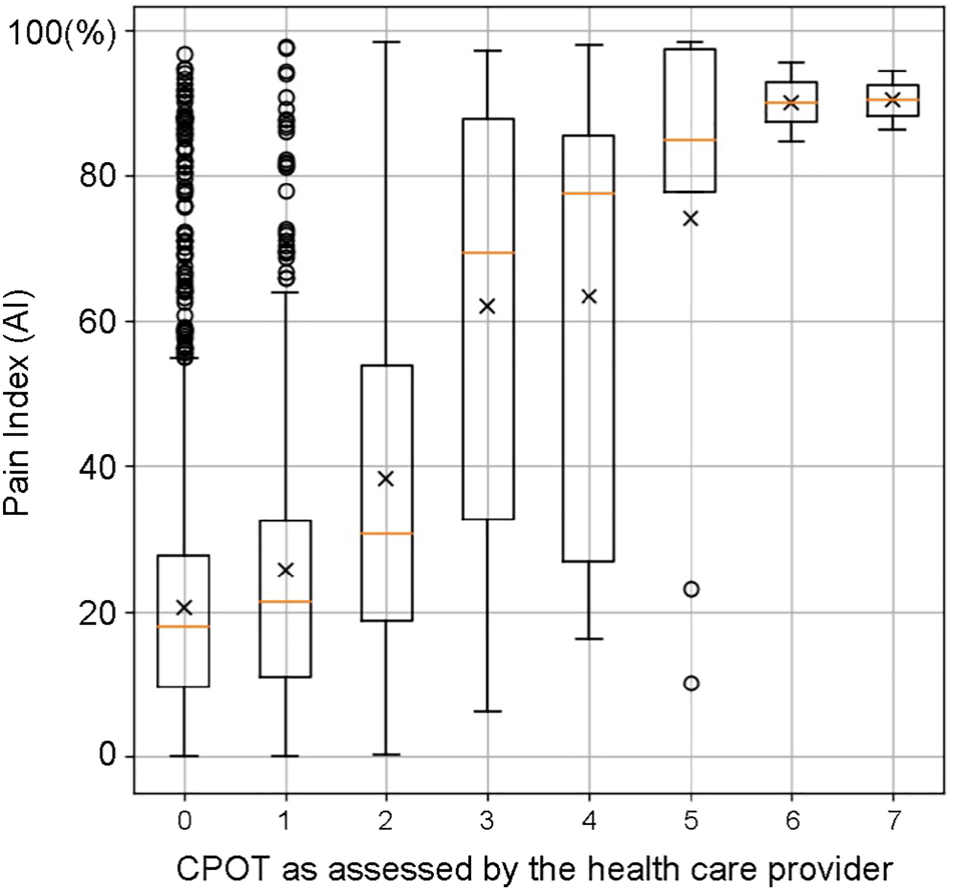
The relationship between the pain index and the CPOT assessed by the healthcare provider. The top and bottom edges of the boxes indicate the quartile range, the horizontal line indicates the median and the crosses indicate the mean. In the CPOT improvement group, the CPOT score decreased by at least 1. The dashed line indicates the median. The upper and lower colored ranges indicate the 75th and 25th percentiles, respectively.

Among the 10,299 eligible patients (Table 1), the CPOT assessment was conducted 117,190 times, and 3925 (3.3%) with a CPOT score of ≥ 3 were judged to have pain. The distribution of the Richmond Agitation-Sedation Scale (RASS) scores, evaluated at approximately the same time, was 0 at 42.7%, ≥ 1 at 8.3%, and ≤ − 1 at 49.0%. The Confusion Assessment Method for the Intensive Care Unit (CAM-ICU) scores indicated that 21.6% of the patients had delirium. The random forest method was used to predict whether a patient had a CPOT score of ≥ 3 points at the time of CPOT evaluation (probability of CPOT being ≥ 3 was defined as pain index), and the AUROC was 0.903, higher than that in the previous study (0.853) (Fig. 1). The importance of the features of the random forest model is shown in Supplementary Table S2. In addition, the generalization performance of the model was verified with an AUROC of 0.873 ± 0.020 over 10 trials with different cross-validation random numbers. The hyperparameters were as follows at the highest accuracy: max_depth: 14; estimates: 17. Accuracy comparisons with other machine learning (logistic regression analysis, LightGBM) performed as subanalyses are shown in Supplementary Fig. S1. The relationship between the pain index and the CPOT assessed by the health care provider is shown in Fig. 2. A trend toward a higher pain index with a higher CPOT was observed.

**Table 1:** Patient characteristics.

Characteristics	Model derivation and validation	Pain visualization test with fentanyl ^a^
Number of patients	10,299	326
Age: year, median (IQR)	65 (73, 52)	67 (74, 52)
Age group: *N* (%)		
20–44	1847 (17.9%)	69 (21.1%)
45–64	3673 (35.7%)	85 (26.0%)
65≤	6007 (58.3%)	130 (39.8%)
Male: *N* (%)	6660 (58.0%)	202 (62.0%)
Days in ICU: days, median (IQR)	1 (3, 1)	4 (9, 1)
Days in ICU: hours, median (IQR)	21.6 (62.6, 17.6)	98.7 (222.2, 24.2)
APACHE II at ICU admission: median (IQR)	13 (17, 10)	16 (20, 13)
Mechanical ventilation: *N* (%)	2479 (24.1%)	88 (27.0%)
Mortality in ICU: *N* (%)	813 (7.9%)	31 (9.5%)
Reason for ICU admission: *N* (%)		
Medical	3985 (38.7%)	111 (34.0%)
Elective operation	5418 (52.6%)	176 (54.0%)
Emergency operation	896 (8.7%)	39 (12.0%)
ICU admission diagnosis: *N* (%)		
Heart	1321 (12.8%)	49 (15.0%)
Heart failure	399 (3.9%)	16 (4.9%)
Acute myocardial infarction	121 (1.2%)	6 (1.8%)
Aorta and vessels	1208 (11.7%)	47 (14.4%)
Kidney	487 (4.7%)	10 (3.0%)
Lung and mediastinum	1155 (11.2%)	44 (13.5%)
Acute respiratory failure	197 (1.9%)	10 (3.1%)
Digestive organs	3154 (30.6%)	85 (26.1%)
Metabolic and endocrine organs	198 (1.9%)	6 (1.8%)
Orthopedics	305 (3.0%)	9 (2.8%)
Genital organs	1053 (10.2%)	16 (4.9%)
Obstetrics	123 (1.2%)	1 (0.3%)
Neurosurgery	654 (6.4%)	5 (1.5%)
Others	1869 (18.1%)	54 (16.6%)
Pain, agitation and delirium assessments		
CPOT: *N* (%)	117,190	22,017
0–2	113,265 (96.7%)	21,437 (97.3%)
≥ 3	3925 (3.3%)	580 (2.6%)
RASS: *N* (%)	136,034	24,267
4	111 (0.1%)	11 (0.0%)
3	600 (0.4%)	117 (0.5%)
2	2899 (2.1%)	450 (1.9%)
1	7727 (5.7%)	1171 (4.8%)
0	58,174 (42.8%)	11,184 (46.1%)
− 1	21,608 (15.9%)	3137 (12.9%)
− 2	13,303 (9.8%)	1940 (8.0%)
− 3	14,663 (10.8%)	2505 (10.3%)
− 4	11,766 (8.6%)	2394 (9.9%)
− 5	5183 (3.8%)	1358 (5.6%)
CAM-ICU: *N* (%)	71,497	11,737
Negative	55,999 (78.3%)	9105 (77.6%)
Positive	15,498 (21.7%)	2632 (22.4%)
Average dosage of sedatives and analgesics (*Number of patients receiving each drug*, %)		
Sedatives		
Dexmedetomidine (µg/kg/h)	0.38 (N: 2380, 21.1%)	0.32 (N: 234, 71.8%)
Propofol (mg/kg/h)	2.05 (N: 1802, 16.0%)	2.68 (N: 221, 67.8%)
Midazolam (mg/kg/h)	0.04 (N: 289, 2.6%)	0.04 (N: 60, 18.4%)
Analgesics		
Fentanyl (µg/kg/h)	1.88 (N: 2878, 25.5%)	1.35 (N: 326, 100.0%)
Hydromorphone	N: 31 (0.3%)	N: 10 (3.1%)
Ketamine	N: 15 (0.1%)	N: 10 (3.1%)
Epidural anesthesia (levobupivacaine)	N: 1235 (12.0%)	N: 0 (0.0%)

*CPOT* critical care pain observation tool, *RASS* richmond agitation-sedation scale, *CAM-ICU* confusion assessment method for the intensive care unit, *ICU* intensive care unit, *IQR* interquartile range.

^a^Patient groups not included in model derivation.

**Figure 3:**
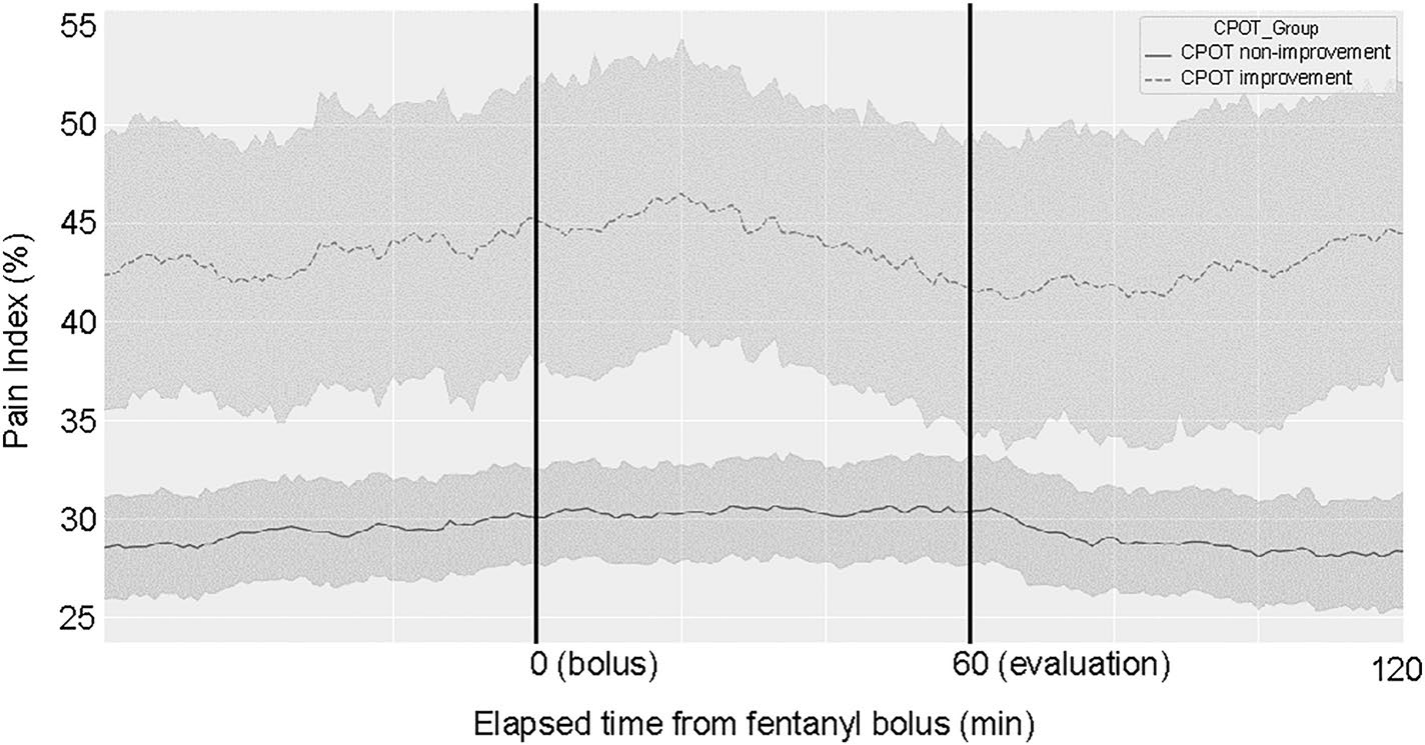
Change over time in pain index with fentanyl bolus administration. The probability of having a CPOT score of ≥ 3 calculated by the artificial intelligence was defined as the pain index, and changes in the pain index were plotted in chronological order before and after fentanyl administration. The time of fentanyl administration was set at 0 min, and the pain index displayed at 60 min was compared between the two groups based on whether the CPOT improved by 1 or more points. The dashed and solid lines in the figure show the median values for the CPOT improvement and no improvement groups, respectively. Statistical evaluation was performed 60 min after the administration of the fentanyl bolus.

The probability of pain (probability of CPOT ≥ 3: pain index, 0–100%) before and after bolus administration of fentanyl was visualized (Fig. 3, see Supplementary Fig. S2 for graphs subdivided by RASS). Patients included were those not used for modeling (Table 1, N = 326). The results showed that the pain index at 1 h after administration was significantly lower in the group whose CPOT improved by more than 1 than that of the group whose CPOT had not improved (at administration: 44.7 ± 27.4; 1 h after administration: 41.5 ± 26.7, *P* = 0.020, Table 2).

**Table 2:** Changes in pain index with fentanyl bolus administration.

CPOT	N	Pain Index (%)		*P* value
		Before fentanyl bolus	Post fentanyl bolus	
All	326	32.3 ± 21.4	31.2 ± 21.6	0.174
Improvement group	58	44.7 ± 27.4	41.5 ± 26.7	0.020*
Non-improvement group	268	29.6 ± 18.9	28.9 ± 19.6	0.632

The CPOT score decreased by at least 1 in the improvement group. The Pain Index indicates the probability of a human-rated CPOT score of ≥ 3. Fisher’s exact probability test was used for statistical analysis.

*CPOT* critical care pain observation tool.

*P < 0.05.

## Discussion

The purpose of this study was to investigate (1) the possibility of improving the prediction accuracy by incorporating deviations from baseline patient vital signs into the prediction model and (2) the concurrence of the predicted AI values with the scores obtained using the CPOT, a conventional objective pain assessment, during analgesic administration. The results showed that (1) pain visualization accuracy corresponded to AUROC 0.902; (2) pain index decreased in tandem only when the CPOT score decreased after a bolus dose of fentanyl.

Pain measurement tools can be divided into self-report tools and behavioral assessment tools. Patients who cannot self-report pain but have observable behaviors must be assessed using behavioral assessment tools^18^. The BPS and CPOT have shown the best validity and reliability to date. CPOT, in particular, has been optimized for use in patients with atypical behaviors, such as those with brain injury^19^. In contrast, neuromuscular blocking agents (NMBAs) are sometimes used in patients with severe breathing problems, such as patients with acute respiratory distress syndrome; however, they chemically paralyze the patient and hinder behavioral assessment^20^. Therefore, periodic discontinuation of NMBA is recommended^21^, and alternative pain assessment methods must be used for the duration of its administration. Although vital signs can be used as a guide for the administration of analgesics and sedatives, they are not recommended as an appropriate indicator of pain due to the large variability that occurs when they are used for pain assessment^22–25^. However, the AI in this study can predict pain with high accuracy despite the use of vital signs and may be applicable even to patients for whom behavioral indicators are not available. Since vital signs are data that are automatically obtained in many patients in ICUs, it may be possible to assess pain automatically and continuously if this AI can be put to practical use.

Recent topics in new objective pain assessment methods, such as the present study, include several electrophysiological tools. Pupil monitoring, a method of assessing pain by recording fluctuations in the pupils in relation to the sympathetic-parasympathetic responses, has been reported to give inconsistent results in critically ill patients^26^. A method of assessing the pupillary response by applying gentle electrical stimulation to the skin has also been developed; however, it can only be used in patients who are under moderate-to-deep sedation for pain^27^. The validity of adding new pain to patients for pain assessment is also questionable. The Analgesia Nociception Index (ANI), which indicates pain on a scale of 0 to 100, has been reported to be particularly useful during dressing changes, with a negative predictive value of 90% using a cut-off of 42.5 points. However, the sensitivity and specificity were 61.4% and 77.4%, respectively, and the number of participants was only a few dozen^28,29^. The Nociception Level (NOL) is a new multiparameter pain assessment system that, similar to the ANI, can display pain on a scale from 0 to 100^30^. In addition to HRV, the device combines photoplethysmography pulse wave amplitude, skin conductance, and body temperature. Pilot studies have shown that NOL is associated with NRS and CPOT during endotracheal suctioning and cuff inflation as well as chest tube removal^31,32^. However, the number of applicable patients in both studies was limited and further validation must be performed in the future. The pain index validated in this study is a new tool to determine pain using vital signs without HRV. It was modeled on data from approximately 10,000 patients and can be varied in parallel with CPOT with an accuracy better than an AUROC of 0.9.

The limitations of this study include its retrospective, single-center design and the possible heterogeneity in general patient background data. In addition, CPOT score was also assessed in patients who were able to communicate to provide continuous pain visualization throughout their ICU stay, and this was used as training data to calculate the scores. The pain index indicates the probability of achieving a CPOT score of ≥ 3. In this situation, there is an immediate risk of accidental tube removal and falls, and the ability to predict these and pain may be useful for patients in ICU. Another problem is that the pain index could not be displayed immediately after ICU admission because the system needs time to establish the baseline as the point at which the patient's condition stabilizes after ICU admission; the pain index is displayed after that point. Furthermore, the fentanyl dose per body weight was not constant in the validation step using the fentanyl bolus dosing data. Lastly, although the pain management protocol was followed, the final dosing decision was made by the charge nurse, and there may have been differences in decision criteria.

In conclusion, this study confirmed that incorporating individual patient baseline data into a previously developed pain visualization model improved the accuracy and treatment follow-up. The next step in the practical application of this model is an open-label, prospective, randomized, controlled trial in a multicenter setting.

## Methods

### Design and study setting

This retrospective observational study was conducted in a single intensive care unit (ICU) at a single institution in Japan. Ethical approval was obtained from Ethics Committee Tohoku University Graduate School of Medicine (Study No. 2022-1-334). Owing to the retrospective design of the study, the requirement for obtaining written informed consent was waived from Ethics Committee Tohoku University Graduate School of Medicine. All methods used in this study were conducted in accordance with the tenets and regulations of the Declaration of Helsinki. The study design was entered into a database (ID: R000047019 UMIN000041179, URL: https://www.umin.ac.jp/ctr/index.htm).

### Participants

Patients admitted to the ICU between October 2016 and March 2019 (1) aged ≥ 20 years with (2) at least five CPOT, RASS, and CAM-ICU assessments and (3) electrocardiography (ECG) and arterial pressure monitoring for at least 30 min were included in the study. Data from the following patients whose vital signs differed significantly from those of the general adult population were excluded: (1) patients who had undergone cardiopulmonary bypass; (2) pregnant patients; (3) patients who had undergone organ transplantation, artificial heart transplantation, extracorporeal membrane oxygenation, and intra-aortic balloon pump surgery; and (4) patients with a do-not-resuscitate order. Data were obtained from the electronic medical records system of the institution (PrimeGaia, Nihon Kohden Corporation, Tokyo, Japan). Patient identification information was not collected. In addition, patients admitted to the ICU between April 2019 and April 2022 who had received a bolus dose of fentanyl (25–100 µg) with vital sign data available for each minute were selected to test the treatment response of a dataset not involved in the creation and validation of these machine learning models.

### Assessment and treatment of analgesia, sedation, and delirium

The CPOT score was used as the training target of the model to assess the pain level of all eligible patients. The CPOT assessments were performed by ICU nurses every 8 h and when obvious pain was observed. The RASS was used to assess the sedation level. Delirium was assessed using the CAM-ICU scale. The RASS, CAM-ICU, and CPOT assessments were used simultaneously by several nurses to ensure agreement among the data recorded by them. In cases of disagreement, the final decision was made by the intensivist. The patients were treated according to our pain management protocol (Supplementary Fig. S3).

### Vital sign data

The heart rate, arterial oxygen saturation, and arterial pressure were recorded every minute. The arterial pressure was continuously monitored via the left or right radial artery. The respiratory rate was measured by electrocardiographic impedance. The noise was removed using the methods described in Supplementary Table S3.

If any of the abovementioned items were applicable to any record, corresponding to a 1-min interval, all vital signs from that record were excluded from the analysis.

### Primary outcome

A CPOT score of 3 was considered positive and a score of 2 was considered negative for pain, and the probability of being positive was calculated using machine learning (pain index). In this study, the predicted probability was defined as the pain index. A grid search was performed on the training data, and the hyperparameters that yielded the highest accuracy for each model were selected. In addition, the harmonic means of the sensitivity, specificity, and AUROC were calculated.

### Machine learning

We used a machine learning algorithm to predict pain when the CPOT score was assessed by a healthcare professional. Random forest, which was reported to be the most accurate algorithm^17^, was used in this study. Two other machine learning methods (logistic regression and LightGBM) were also used for supplementary analysis. The characteristics of each machine learning method are shown in Supplementary Table S4. The following explanatory variables were included: arterial pressure (systolic, mean, and diastolic), heart rate, respiratory rate, sex, stratified age (20–44, 45–64, and 65+ years), and the RASS score. The CAM-ICU score was used for exploratory analysis but not for building the final model. Information processing was conducted to reflect the individual differences in the vital signs of each patient in the model.

#### Noise canceling and oversampling

The noise in the vital sign data was removed according to the requirements listed in Supplementary Table S3. Owing to the relatively small number of pain-positive patients, the positive group was oversampled using adaptive synthetic sampling. The building process illustrated in Supplementary Fig. S4 was followed to build the subsequent model to avoid the effects of overfitting. Tuning of hyperparameters was targeted for n_estimator and max_depth. See Supplementary Fig. S5 for details.

#### Baseline evaluation

Baselines were obtained for each patient and adjusted as shown below (Supplementary Fig. S6):

1. Hourly records of patients admitted to the ICU were extracted.
2. Approximately 90% of the hourly data were evaluated for availability and two-tailed linear completion was performed.
3. The hourly data were divided into three chronological orders. Friedman tests were performed to evaluate statistically significant differences among the three age groups. The condition was considered unstable if a significant statistical difference was observed, and the condition was returned to condition (ii) after 20 min.
4. Variability (standard deviation/mean) was calculated to ensure that the condition did not deviate from the threshold values established for each type of vital sign in previous studies. The condition was considered unstable if the threshold was exceeded, and the patient was moved to (ii) after 20 min.
5. The 5-min moving average during this period was used as the baseline.

#### Calculation of the estimated probability of pain (pain index)

The estimated probability of pain during the CPOT assessment was also calculated. This probability was defined as the pain index. Vital signs from 1 h before the evaluation were extracted. After linear interpolation in both directions for the interval where more than 90% of the data existed in 1 h, the area over or under the baseline curve was obtained, and the difference from the baseline was calculated. The calculation methods and formulas for these characteristics are shown in Supplementary Fig. S7. Lastly, the age group, sex, and RASS scores were linked to the training data.

#### Evaluation of treatment response (visualization)

The pain index was visualized using a new dataset that was not used to build and validate the model. The patients who received a bolus dose of fentanyl and for whom vital data could be obtained each minute were divided into two groups according to CPOT score improvement by more than 1. The transition was graphed for each group. Other analgesics (ketamine and morphine) had only temporary and limited use and were analyzed for periods when these drugs were not administered.

### Statistical analysis

Data analysis was performed using JMP v15 (SAS Institute Inc., Cary, NC, USA). Normally distributed data were reported as mean ± standard deviation, and non-normally distributed data were reported as median and interquartile range. The AUROC was used to compare the accuracy and was classified as low (0.5–0.7), moderate (0.7–0.8), and high (≥ 0.8).

### Supplementary Information

Supplementary Information.

## Data Availability

The datasets generated and/or analyzed during the current study are available in the UMIN-ICDR repository at http://www.umin.ac.jp/icdr/index-j.html, with permission from the authors.
