# Supplementary material for: Continuous visualization and validation of pain in critically ill patients using artificial intelligence: a retrospective observational study
Source: Sci Rep. 2023 Oct 14;13:17479. doi: 10.1038/s41598-023-44970-2 (PMC10576770; doi:10.1038/s41598-023-44970-2)
Supplement: Supplementary file 1 — Supplementary Information. [file 41598_2023_44970_MOESM1_ESM.docx]

**Continuous Visualization and Validation of Pain in Critically Ill Patients Using Artificial Intelligence: A Retrospective Observational Study**

Naoya Kobayashi^1*^, Kazuki Watanabe^2^, Hitoshi Murakami^2^, Masanori Yamauchi^1^

^1^Department of Anesthesiology and Perioperative Medicine, Tohoku University Graduate School of Medicine, Sendai, Miyagi, Japan

^2^Hitachi Solutions East Japan, Ltd, Sendai, Miyagi, Japan

^*^Corresponding author: Naoya Kobayashi

E-mail: [naoya.kobayashi.a4@tohoku.ac.jp](mailto:naoya.kobayashi.a4@tohoku.ac.jp)

**Supplementary Material**

**Supplementary Table S1.** Critical Care Pain Observation Tool (CPOT)

| Indicator | Description | Score |  |
| --- | --- | --- | --- |
| Facial expression | No muscular tension observed | Relaxed, neutral | 0 |
|  | Presence of frowning, brow lowering, orbit tightening, and levator contraction | Tense | 1 |
|  | All of the above facial movements plus eyelids tightly closed | Grimacing | 2 |
| Body movements | Does not move at all (does not necessarily mean absence of pain) | Absence of movements | 0 |
|  | Slow, cautions movements, touching or rubbing the pain site, seeking attention through movements | Protection | 1 |
|  | Pulling tube, attempting to sit up, moving limbs/thrashing, not following commands, striking at staff, trying to climb out of bed | Restlessness | 2 |
| Muscle tension evaluation by passive flexion and extension of upper extremities | No resistance to passive movements | Relaxed | 0 |
|  | Resistance to passive movements | Tense, rigid | 1 |
|  | Strong resistance to passive movements, inability to complete them | Very tense or rigid | 2 |
| Compliance with the ventilator (intubated patients) | Alarms not activated, easy ventilation | Tolerating ventilator or movement | 0 |
|  | Alarms stop spontaneously | Coughing but tolerating | 1 |
|  | Asynchrony: blocking ventilation, alarms frequently activated | Fighting ventilator | 2 |
| OR Vocalization | Talking in normal tone or no sound | Talking in normal tone or no sound | 0 |
|  | Sighing, moaning | Sighing, moaning | 1 |
|  | Crying out, sobbing | Crying out, sobbing | 2 |

Critical Care Pain Observation Tool referred to literature number [11]. This evaluation method is an objective way to assess pain in patients in ICU who are unable to communicate.

ICU, intensive care unit

**Supplementary Table S2.** Basic statistics of features and their importance in random forest models

| Features | CPOT less than 2 | CPOT of 3 or higher | P value |  | Importance |
| --- | --- | --- | --- | --- | --- |
| Number of records | 34541 | 953 |  |  |  |
| Age: year, median (IQR) | 65.0 (52.0, 73.0) | 65.0 (54.0, 74.0) | 0.223 |  |  |
| Male: N(%) | 2172(56.8%) | 215(61.8%) | 0.078 |  | 0.007 |
| Age group: N(%) |  |  | 0.362 |  | 0.019 |
| 20-44 | 626(16.4%) | 48(13.8%) |  |  |  |
| 45-64 | 1247(32.7%) | 123(35.3%) |  |  |  |
| 65≤ | 1944(50.9%) | 177(50.9%) |  |  |  |
| RASS: N(%) |  |  | < 0.001 | ** | 0.386 |
| 4 | 1(0%) | 4(1.1%) |  |  |  |
| 3 | 5(0.1%) | 28(8.0%) |  |  |  |
| 2 | 42(1.1%) | 119(34.1%) |  |  |  |
| 1 | 247(6.5%) | 92(26.4%) |  |  |  |
| 0 | 3185(83.4%) | 98(28.2%) |  |  |  |
| -1 | 197(5.2%) | 4(1.1%) |  |  |  |
| -2 | 58(1.5%) | 1(0.3%) |  |  |  |
| -3 | 45(1.2%) | 0(0%) |  |  |  |
| -4 | 29(0.8%) | 2(0.6%) |  |  |  |
| -5 | 8(0.2%) | 0(0%) |  |  |  |
| Vital sign data  (Integral difference from stable period, from each record to 60 minutes before median (IQR)) |  |  |  |  |  |
| Diastolic arterial pressure + (mmHg) | 115 (-80, 572) | 140 (-75, 592) | 0.312 |  | 0.039 |
| Diastolic arterial pressure – (mmHg) | -61 (-117, 90) | -76 (-143, 46) | < 0.001 | ** | 0.049 |
| Systolic arterial pressure + (mmHg) | 288 (-21, 931) | 336 (-4, 942) | 0.167 |  | 0.046 |
| Systolic arterial pressure – (mmHg) | -31 (-69, 182) | -33 (-84, 216) | 0.130 |  | 0.049 |
| Mean arterial pressure + (mmHg) | 484 (-6, 1527) | 767 (75, 1780) | < 0.001 | ** | 0.059 |
| Mean arterial pressure – (mmHg) | -19 (-53, 326) | -26 (-63, 240) | < 0.001 | ** | 0.047 |
| Pulse rate + (per minutes) | -14 (-51, 276) | 19 (-45, 471) | < 0.001 | ** | 0.056 |
| Pulse rate – (per minutes) | 129 (-24, 790) | 23 (-33, 624) | < 0.001 | ** | 0.047 |
| Heart rate + (per minutes) | -13 (-72, 291) | 0 (-78, 409) | 0.223 |  | 0.045 |
| Heart rate – (per minutes) | 128 (-30, 787) | 82 (-38, 845) | 0.193 |  | 0.047 |
| Respiratory rate + (per minutes) | 40 (-37, 247) | 52 -(17, 262) | 0.009 | ** | 0.059 |
| Respiratory rate – (per minutes) | 1 (-22, 112) | 1 (-19, 110) | 0.643 |  | 0.046 |

The data are shown after all the processes shown in the method have been completed. The "+" and "–" after each parameter indicate the upward or downward variation from the previous value, respectively. For example, in the upward direction, a downward variation from the previous value is calculated as 0. The difference between the cumulative values for 60 minutes during the stability period and the cumulative values from each record to 60 minutes before was determined. Negative values in the table indicate that the variation was even smaller than in the stability period. Train AUROC: 0.988; Test AUROC: 0.975; CPOT, Critical Care Pain Observation Tool; IQR, interquartile range; RASS, Richmond Agitation-Sedation Scale.

**Supplementary Table S3.** Noise cancelation method for arterial blood pressure data

| Factors | Requirements |
| --- | --- |
| Systolic pressure | < 35, 300 < |
|  | < 80 changes from previous record |
|  | < 40 changes were measured twice or more consecutively |
| Diastolic pressure | < 20 |
| Systolic and diastolic pressure | (Systolic pressure) - (Diastolic pressure) < 15 |
| Heart rate | < 0, 250 < |
| Respiratory rate | < 3, 81 < |

**Supplementary Table S4.** Brief schema of the machine learning methods applied in this study

|  | Random forest | Light gradient boosting machine (LightGBM) | Logistic regression |
| --- | --- | --- | --- |
| Explanation | A method of constructing numerous decision trees and carrying out a majority vote. It requires a large amount of data but can make highly accurate predictions. | A method of multiple decision tree analysis using a method called gradient boosting ^a^. | Calculates the class membership probability for two categories by fitting the log odds and explanatory variables to a model |
| Speed | **Moderate**  Depending on the number of data and the number of dimensions chosen to build a single tree, it is often faster than support vector machine when the data size is large. | **Low**  Computational cost is high, depending on the amount of data, to find the dividing factors and criteria to minimize the error. | **High**  It utilizes stochastic gradient descent, and once the gradient is obtained, it can quickly find the solution and be easily applied to large datasets. |
| Accuracy | **High**  When solving simple problems with a small amount of data, the accuracy is low owing to unavailability of data required for the large number of calculations. In contrast, when the data size is large, the accuracy is high, and overlearning is unlikely to occur. | **High**  Using a method called gradient boosting ^a^, multiple decision tree analysis is more accurate. | **Low**  Performs calculations by applying a sigmoidal function to the output of a multiple regression. If the target variable tends to be in a single direction, the probability of correct classification is high. However, if there is a skewed class bias in the feature space, classification may be difficult to perform. |
| Calibration | **Easy**  Only the depth and number of decision trees need to be adjusted. Further, regularization and standardization of the training data are not required. | **Difficult**  There are many items to consider, as the "number of leaves (num_leaves)," "minimum number of data in one leaf (min_data_in_leaf)," and "depth of hierarchy (max_depth)" of the decision tree must be tuned while balancing model accuracy and overlearning. | **Easy**  Only the adjustment of slack variables is necessary. Further, regularization and standardization of the training data are not required. |

The table presents a comparative representation of the three machine learning methods. ^a^: Boosting" is a method of improving accuracy by performing a decision tree analysis from given data, then weighting the data that failed to predict correctly, performing the decision tree analysis again, and repeating the process. Furthermore, instead of weighting the data, "gradient boosting" is a method that calculates the error between the forecast value and the actual value and uses the error to learn from the decision tree. As with boosting, the accuracy is improved by repeating the learning process for the errors.

**
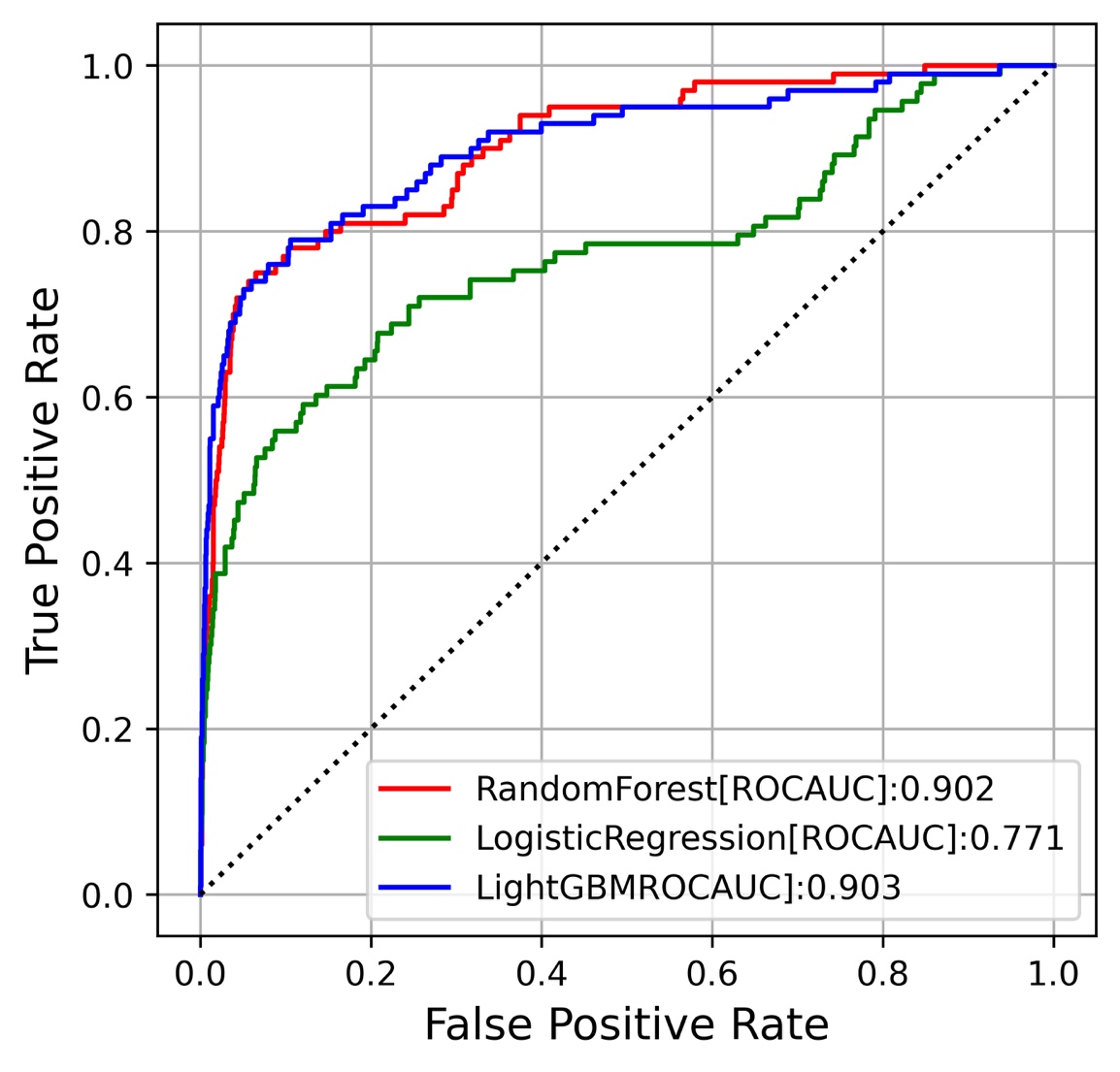
**

**Supplementary Figure S1**. Comparison of Machine Learning Accuracy. The x-axis and y-axis represent the negative sensitivity and specificity in the ROC curve, respectively. The accuracy of the test depends on the ability of the machine learning model to correctly determine whether the CPOT score is <2 or >3. The accuracy is expressed by AUROC, where a range of 1 indicates a perfect test and a range of 0.5 indicates an inconclusive test. In the random forest, the sensitivity and specificity were 73.0% and 94.5%, respectively. In the logistic regression, the sensitivity and specificity were 72.0% and 71.6%, respectively. For LightGBM, the sensitivity and specificity were 62.0% and 97.7%, respectively.

**A)** CPOT score improved by more than 1

**
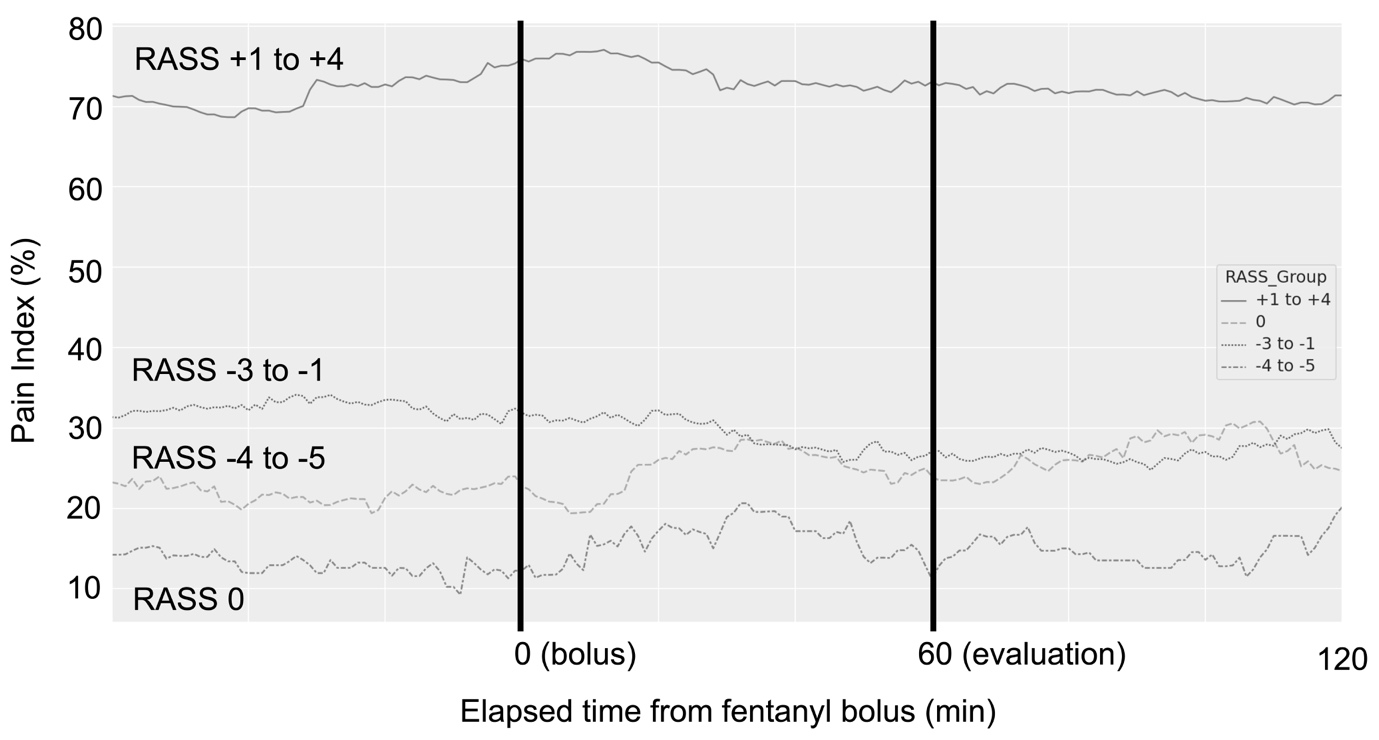
**

**B)** CPOT score had not improved

**
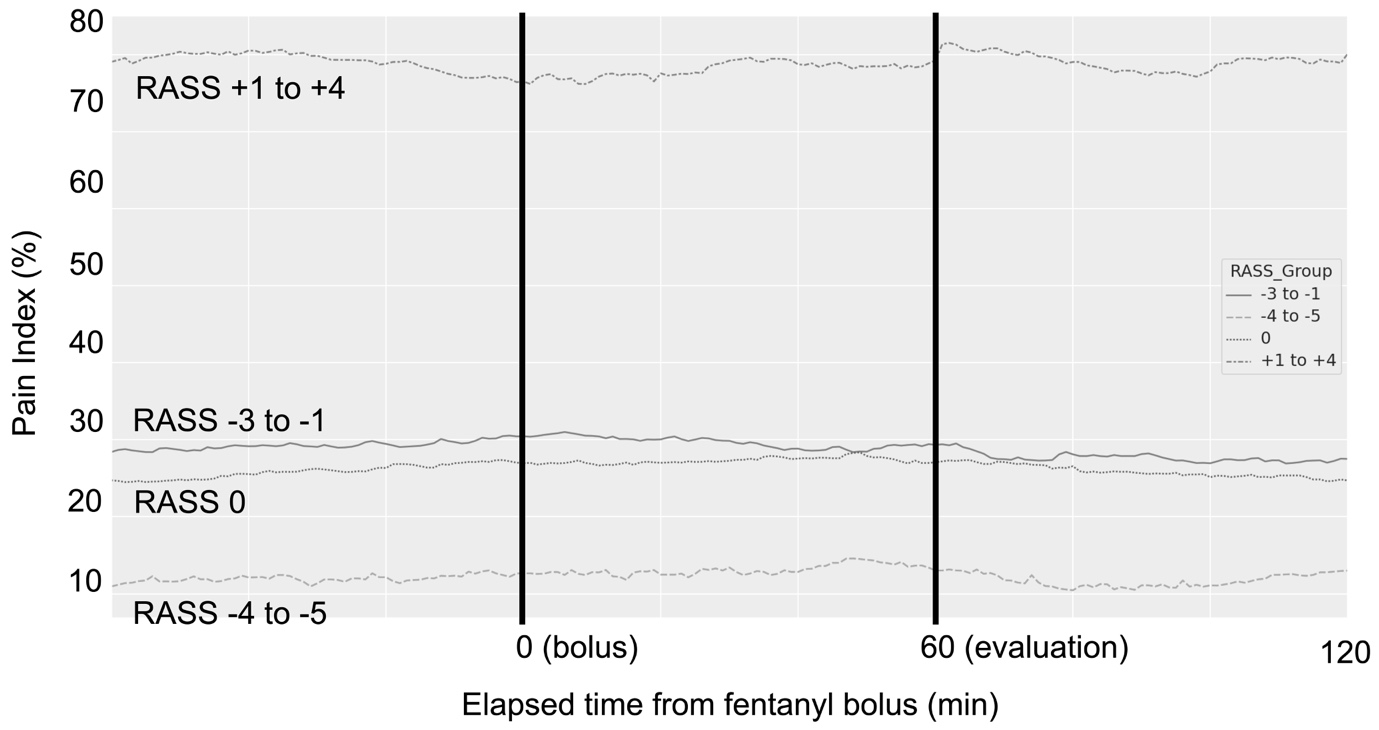
**

**Supplementary Figure S2**. Pain Index movement after fentanyl bolus administration classified by RASS. A: CPOT score improved by more than 1. B: CPOT score had not improved. RASS, Richmond Agitation-Sedation Scale; CPOT, critical care pain observation tool

**
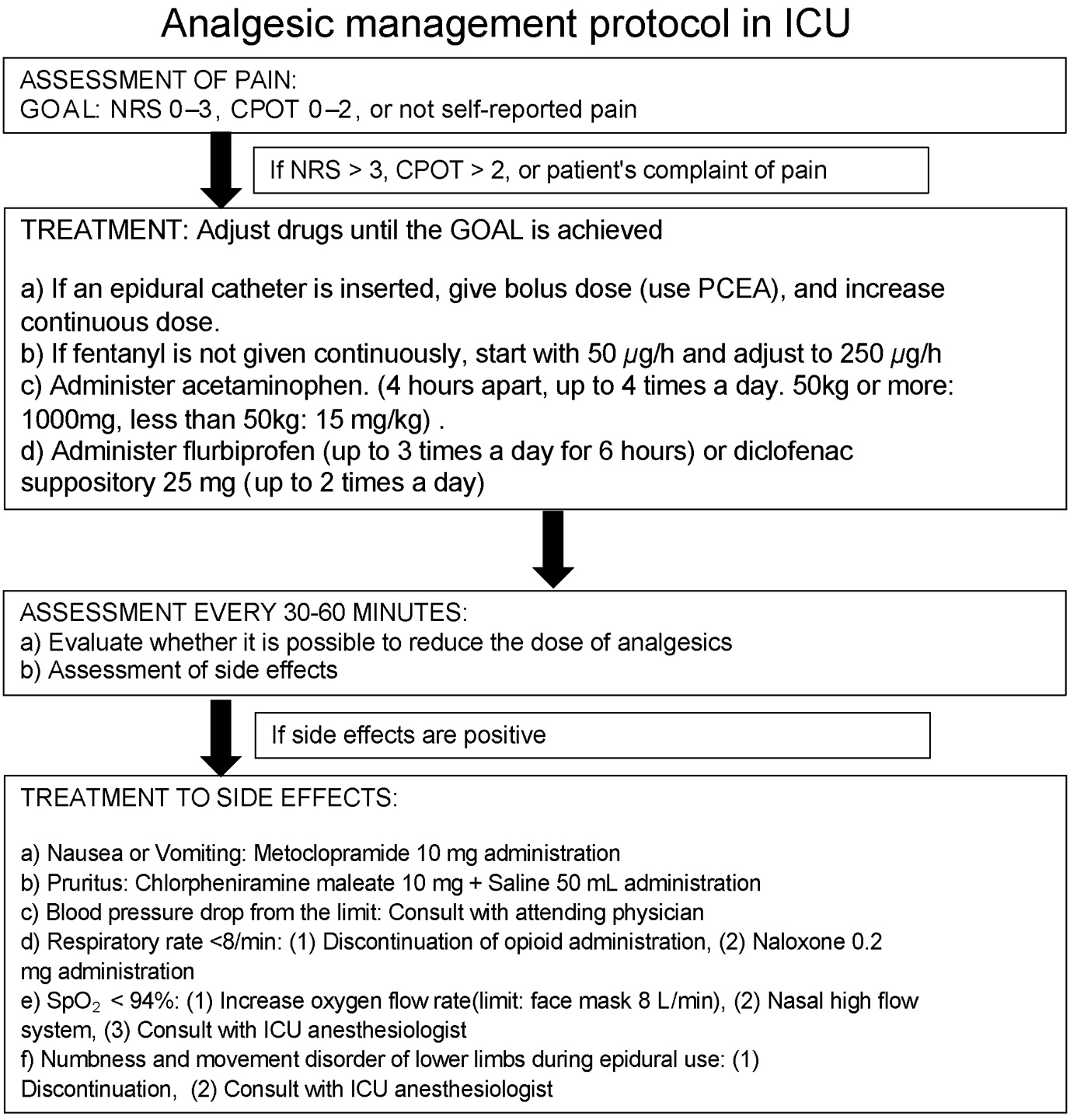
**

**Supplementary Figure S3**. Analgesic management protocol. All eligible patients were managed for pain according to the protocol. Flurbiprofen is not to be administered to patients with renal dysfunction, asthma, or other contraindicated diseases or patients with severe disease above a specific level in treatment d). CPOT assessment was performed by the intensive care nurses every 8 hours and when obvious pain was observed.

NRS, numerical rating scale; CPOT, critical care pain observation tool; PCEA, patient-controlled epidural analgesia.


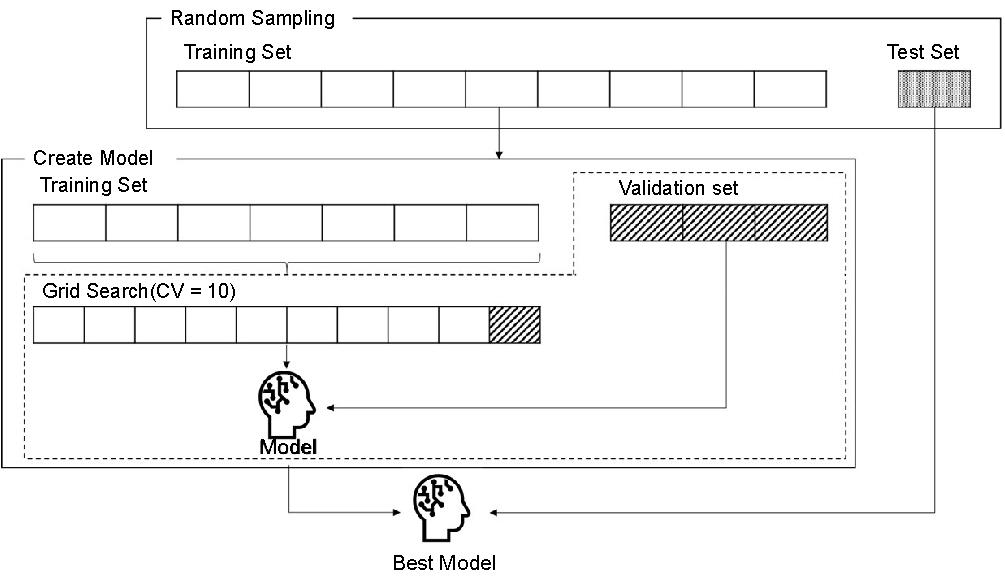


**Supplementary Figure S4.** Modeling and validation to avoid overfitting. The model was created and validated using the following procedure to avoid overfitting. (i) All patients used for modeling were randomly divided into two groups, 90%:10% (N= 9269:1030). (ii) The 90% group was further divided into 70%:30% (N= 6488:2781). (iii) The 70% group was cross-validated 10 times to create a model, which was validated using the 30% group. (iv) The model was cross-validated again using the 10% group from (i). (v) Steps 1) through 4) were repeated 10 times. If overfitting occurs, the accuracy of step (iv) will be very low.


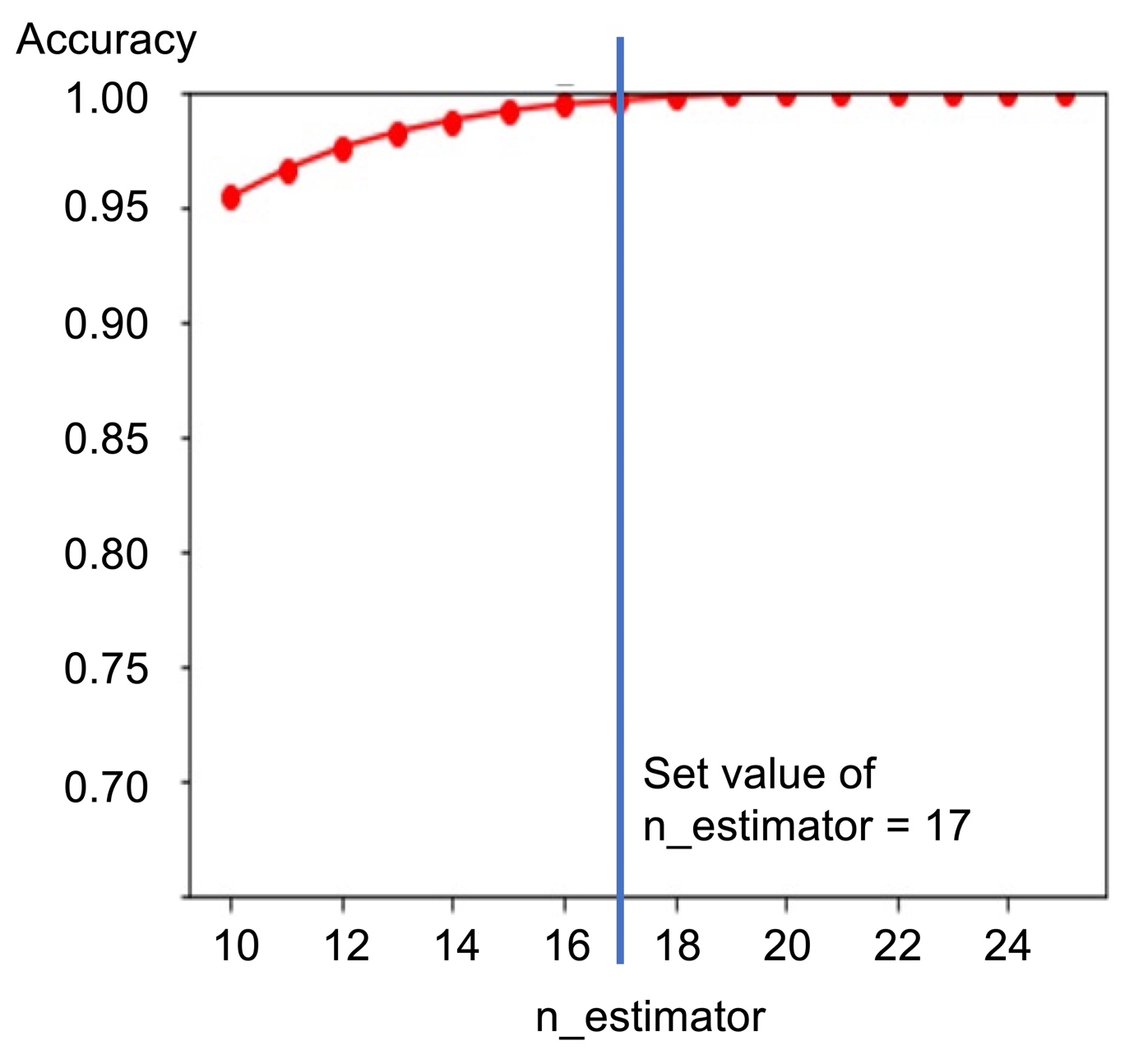


**Supplementary Figure S5.** Tuning of hyperparameters on n_estimator. GridSearch was performed with n_estimator and max_depth set from 10 to 100 (in increments of 10) and max_depth from None to 30 (in increments of one). The results showed that n_estimator and max_depth of 10 to 20 and None for max_depth showed high accuracy. We also searched for the optimal value of n_estimator using the validation curve (see figure). The results showed that there was little change from n_estimator=17, so only n_estimator=17 was set, and validation was performed.


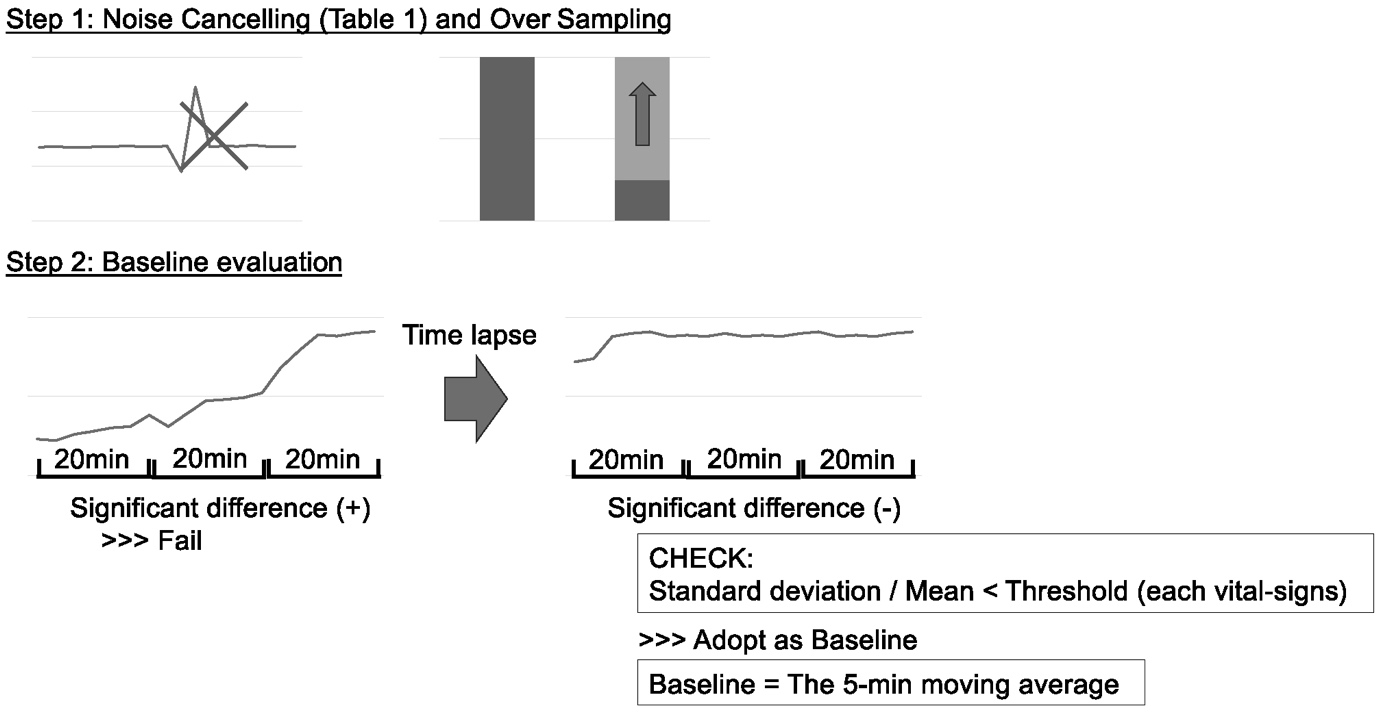


**Supplementary Figure S6.** Baseline evaluation.


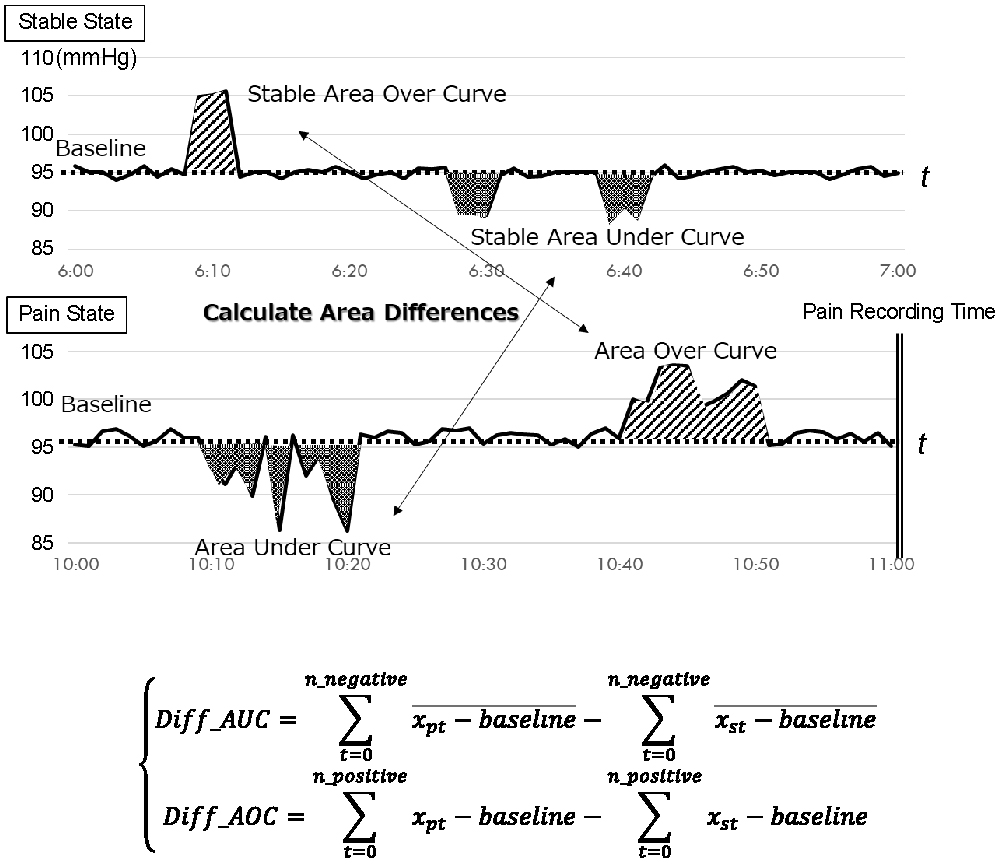


**Supplementary Figure S7.** Vital signs, comparison examples, and formulas. Vital sign data were used as predictors of deviation from baseline and calculated for each patient.
